# Fit to dwell in many places – The growing diversity of intracellular *Salmonella* niches

**DOI:** 10.3389/fcimb.2022.989451

**Published:** 2022-08-18

**Authors:** Chak Hon Luk, Jost Enninga, Camila Valenzuela

**Affiliations:** ^1^ Institut Pasteur, Unité « Dynamique des interactions hôte-pathogène » and CNRS UMR3691, Université de Paris Cité, Paris, France; ^2^ Host-Pathogen Interactions in Tuberculosis Laboratory, The Francis Crick Institute, London, United Kingdom

**Keywords:** *Salmonella*, host cell, epithelial cells, fibroblasts, macrophages, host-pathogen interactions

## Abstract

*Salmonella enterica* is capable of invading different host cell types including epithelial cells and M cells during local infection, and immune cells and fibroblasts during the subsequent systemic spread. The intracellular lifestyles of *Salmonella* inside different cell types are remarkable for their distinct residential niches, and their varying replication rates. To study this, researchers have employed different cell models, such as various epithelial cells, immune cells, and fibroblasts. In epithelial cells, *S.* Typhimurium dwells within modified endolysosomes or gains access to the host cytoplasm. In the cytoplasm, the pathogen is exposed to the host autophagy machinery or poised for rapid multiplication, whereas it grows at a slower rate or remains dormant within the endomembrane-bound compartments. The swift bimodal lifestyle is not observed in fibroblasts and immune cells, and it emerges that these cells handle intracellular *S.* Typhimurium through different clearance machineries. Moreover, in these cell types *S*. Typhimurium grows withing modified phagosomes of distinct functional composition by adopting targeted molecular countermeasures. The preference for one or the other intracellular niche and the diverse cell type-specific *Salmonella* lifestyles are determined by the complex interactions between a myriad of bacterial effectors and host factors. It is important to understand how this communication is differentially regulated dependent on the host cell type and on the distinct intracellular growth rate. To support the efforts in deciphering *Salmonella* invasion across the different infection models, we provide a systematic comparison of the findings yielded from cell culture models. We also outline the future directions towards a better understanding of these differential *Salmonella* intracellular lifestyles.

## Introduction

**Figure 1:**
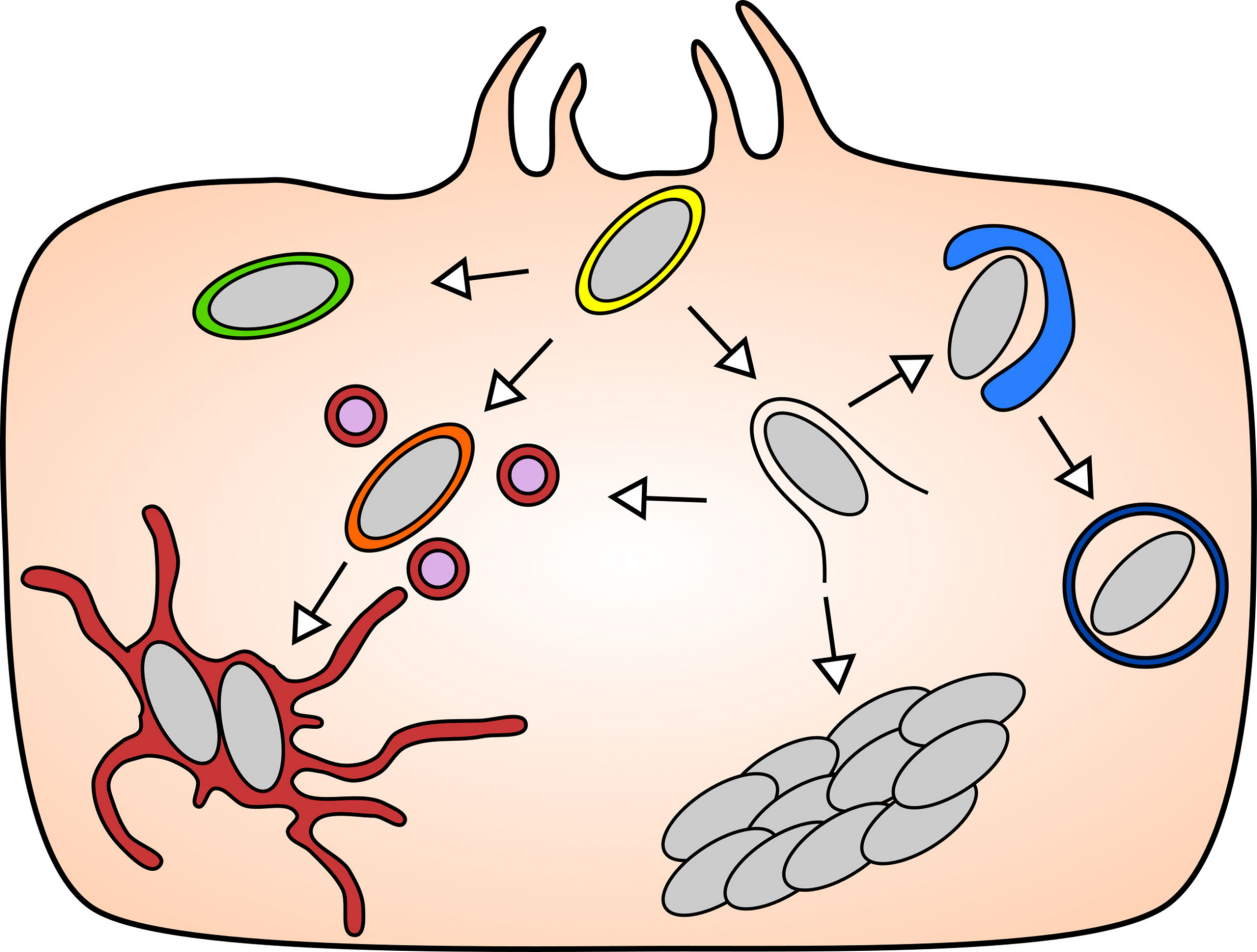
The diverse intracellular lifestyles of *Salmonella*: In the mammalian model systems, *S*. Typhimurium has been found to adopt a range of lifestyles of distinct features that are remarkable for their subcellular localizations and replication rates. Upon entry into the host cell (yellow), *S*. Typhimurium can opt to remain either inside the SCV, or to enter the host cytosol by rupturing the SCV. The vacuolar *S*. Typhimurium give rise to replicative (maroon) and non-replicative (green) subpopulation, while the cytosolic *S*. Typhimurium can be targeted by host autophagy (blue), or they hyper-replicate (bacterial cluster) or re-enter the SCV via repair of the damaged SCV (orange).

Salmonellosis is caused by *Salmonella enterica* subsp. *enterica* that includes both typhoidal and non-typhoidal *Salmonella* (NTS) serovars. Infection leads to different symptoms encompassing enteric fever, self-limiting gastroenteritis, septicemia, focal infections and, in the case of some typhoidal strains, an asymptomatic carrier state. NTS-caused gastroenteritis is generally self-resolving in healthy adults, but it can also cause systemic infection in immunosuppressed humans as well as very young and older individuals. *Salmonella* pathogenicity has been mostly studied by using *Salmonella enterica* serovar Typhimurium (hereafter referred as *S*. Typhimurium). It starts in the intestine, and then it can spread to other organs, such as the spleen or liver. A hallmark of this pathogen is its capacity to actively induce its internalization in a wide range of host cells and avoiding destruction by professional phagocytic cells. The interaction with the different host cells leads to the formation of specific intracellular niches with diverse growth rates and clearance efficacies (see **Figure 1**); all of which will be compared in this review.

## Epithelial model cells for the study of early *Salmonella* infection

During early challenge of the host by *S*. Typhimurium, the intestinal epithelium is the first susceptible tissue encountered within the host gastrointestinal tract. It is mainly constituted of enterocytes to absorb nutrients, and other interspersed cell types, such as Goblet cells, Microfold cells, enteroendocrine cells, and Paneth cells. To understand the consecutive events in the disease progression in epithelial tissues, a number of *in vitro* cell culture models have been adopted as proxies for the endogenous infection conditions of *S*. Typhimurium. These cell culture models include HeLa (cervical adenocarcinoma epithelial cell), Caco-2 (colon carcinoma epithelial cell), HT-29 (colon colorectal adenocarcinoma), T84 (colon carcinoma from lung metastasis) and MDCK (transformed dog kidney cells). The above-mentioned cell lines are selected as model systems based on two primary criteria: the representation of key biological features during an infection and the compatibility towards the experimental setup and procedures (**Table 1**). HeLa is a human-derived cervical carcinoma epithelial cell, a well-characterized and widely used cell culture model, easy for genetic manipulation including transfection, knock-in and knockout lines; it is also highly amenable for biochemical and imaging approaches. However, HeLa is derived from cervical tissue, a non-native site of *S*. Typhimurium infection. Caco-2 and HT-29 are epithelial cell lines derived from human colon, which are capable of differentiating into polarized epithelial monolayers that closely represent the microvilli structures of the gut epithelium. Mucus-secreting HT-29-MTX could produce a mucus layer on the cell surface that facilitates endogenous bacterial attachment (Gagnon et al., 2013). A limitation of the Caco-2 and HT-29 models is their combination with the widely used trans-wells for growing them into non-permeable polarized monolayers; those sample holders are not compatible with live imaging and certain imaging techniques, such as those requiring large numerical aperture objectives or high-resolution imaging. The T84 cells are taken from a colon carcinoma metastasis isolated from the lung that share similarities with Caco-2 and HT-29. Comparing the morphology of polarized colonic epithelial cells, Caco-2 form microvilli that resembles small intestine epithelium while T84 form a layer reminiscent of colonic epithelium (Devriese et al., 2017). MDCK is a canine-derived kidney epithelial cell line, capable of forming a polarized monolayer, but it does not form tight cell-cell connections that can be measured by transepithelial electrical resistance. Researchers typically use more than one of the above cell lines in cellular studies to verify their hypotheses of the endogenous infected conditions.

**Table 1:** Cell lines commonly used for *Salmonella* analysis of intracellular lifestyles and main references exploring specific intracellular niches.

	Cell line	Key features	Vacuolar	Cytosolic	Dormant/Persistant
**Epithelial cells**	HeLa	Widely used, easy genetic manipulation and imaging	(Beuzón et al., 2000; Schroeder et al., 2010; Krieger et al., 2014; Santos et al., 2015; Liss et al., 2017; Stévenin et al., 2019)	(Knodler et al., 2010; Finn et al., 2017; Powers et al., 2021)	(Luk et al., 2021)
	Caco-2	Capable of differentiating into polarized epithelial monolayer, similar to small intestinal enterocytes	(Stévenin et al., 2019)	(Knodler et al., 2010; Knodler et al., 2014)	(Luk et al., 2021)
	HT-29	Capable of differentiating into polarized epithelial monolayer. HT-29-MTX produce mucus layer	–	(Knodler et al., 2014)	–
	T84	Capable of differentiating into polarized epithelial monolayer, reminiscent of colonocytes	–	(Knodler et al., 2014)	–
**Fibroblasts**	MEF	Widely used, easy genetic manipulation, lower antibacterial response	(Kreibich et al., 2015)	(Thurston et al., 2016a; Thurston et al., 2016b)	(Hernández et al., 2022)
	3T3	Strong antibacterial response	–	(Thurston et al., 2016b; Thurston et al., 2016a)	(Luk et al., 2021)
	NRK-49F	Lower cytotoxicity upon bacterial infection	(Núñez-Hernández et al., 2014; López-Montero et al., 2016; Castanheira et al., 2017)	–	(Núñez-Hernández et al., 2014; López-Montero et al., 2016)
	BJ-5ta	Human origin, provides better representation of human system	(Aiastui et al., 2010)	–	–
**Immune cells**	RAW264.7	Murine macrophages. Easy manipulation but lack certain inflammatory cascades	(Drecktrah et al., 2006; Krieger et al., 2014; Diacovich et al., 2017; Günster et al., 2017)	–	(Helaine et al., 2010)
	J774A.1	Murine macrophages. Presents some differences in inflammatory cascades compared with RAW264.7		(Lau et al., 2019)	
	BMDM	Close representation of an endogenous macrophage	(Rosenberg et al., 2021)	(Thurston et al., 2016b)	(Helaine et al., 2014)
	THP-1	Monocytes that can be differentiated into macrophage-like cells	–	(Shi et al., 2014)	(Luk et al., 2021)
	hMDM	Large genetic background diversity due to differences in donnors	(Lathrop et al., 2015)		

Generally, it has been described that *S*. Typhimurium is capable of entering the non-phagocytic epithelial enterocyte monolayer through manipulation of the host cell cytoskeleton with the aid of a Type III Secretion System (T3SS) and its injecting bacterial effectors (LaRock et al., 2015). On the other hand, entry paths independent of the T3SS effectors have been investigated, for example through the interaction of the bacterial outer membrane protein Rck with the host receptors, such as the EGF receptor (Velge et al., 2012). Furthermore, for a long time it was thought that *S*. Typhimurium entered via the main mechanism of T3SS-1 dwells inside a macropinosome, but analysis at the single bacterium level has shown that the active entry of *S*. Typhimurium into epithelial cells is inside a tight vacuole, coined *Salmonella*-containing compartment (SCC) (Fredlund et al., 2018). Passive entry inside macropinosomes is also possible, a phenomenon mediated by other “helper bacteria” during infection, an effect that is more prevalent at higher multiplicity of infection (Fredlund et al., 2018). The SCC is then subjected to a dynamic size change to generate the *Salmonella*-containing vacuole (SCV) within epithelial cells. Formation of spacious vacuole-associated tubules (SVATs) shrinks the SCV while the fusion with macropinosomes increases its size (Stévenin et al., 2019). These two events result in a dynamic size-regulation of SCV stability controlled by fusion and extraction of membrane.

**Figure 2:**
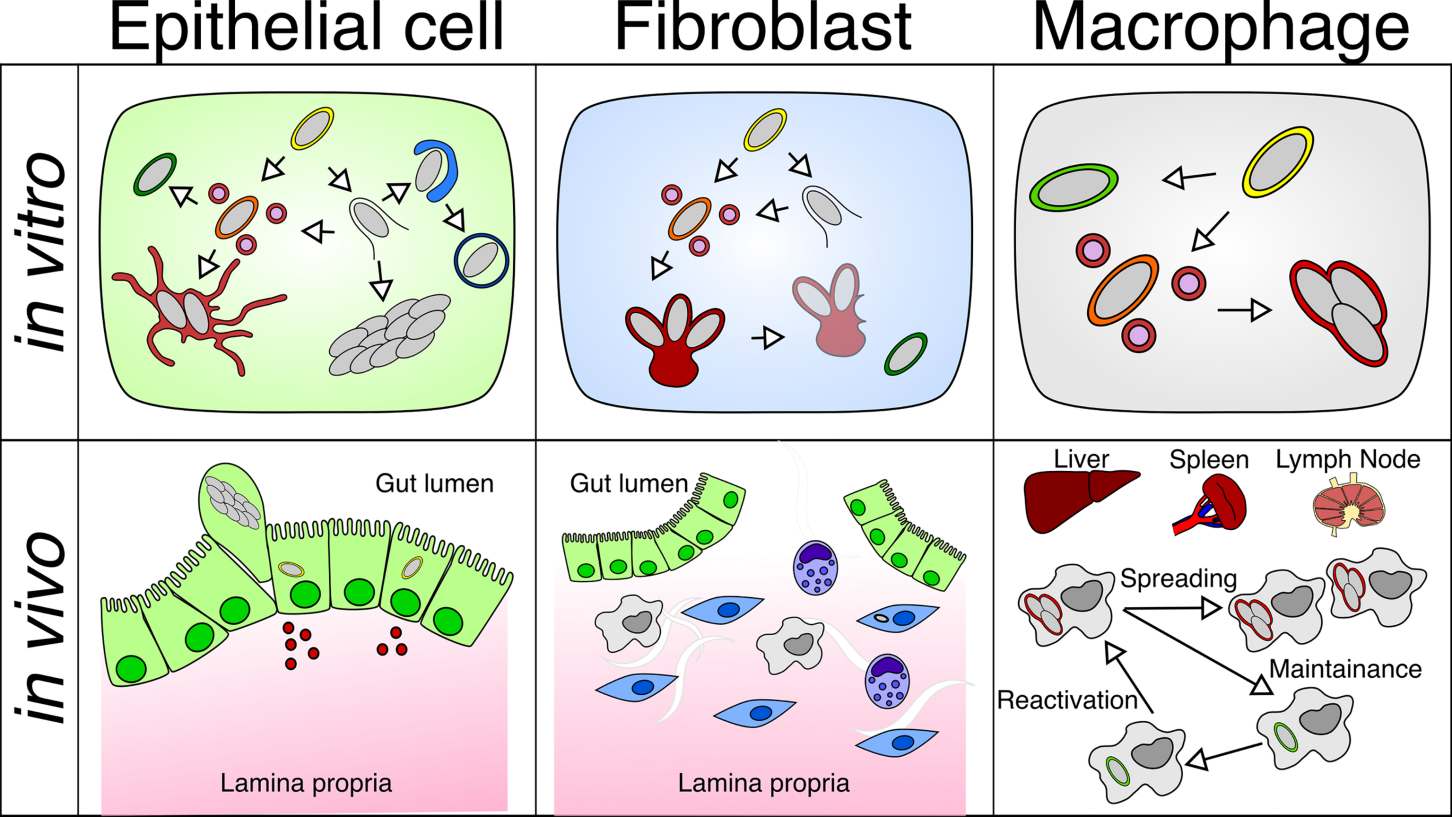
Differential behavior of *Salmonella* within different cell types *in vitro* or *in vivo*: *S*. Typhimurium adopts different intracellular lifestyles in epithelial cells, fibroblasts and macrophages under *in vitro* and *in vivo* conditions. (Top left) *S*. Typhimurium propagates inside the SCV or the host cytosol of the epithelial cell, which is linked to different replication rates. (Top middle) *S*. Typhimurium only replicates within the aggresome, and *S*. Typhimurium detached from the aggresome can persist in fibroblasts. (Top right) *S*. Typhimurium undergoes a replicative or dormant lifestyle inside the SCV of macrophages. (Bottom left) Fast replicating *S*. Typhimurium leads to the activation of inflammatory pathways in the epithelium, which causes the extrusion of epithelial cells harboring fast-growing *S*. Typhimurium and the release of inflammatory cytokines. (Bottom middle) *S*. Typhimurium resides in a small fraction of fibroblasts in the gut lamina propria. (Bottom right) *S*. Typhimurium are found in macrophages residing in the liver, spleen, and mesenteric lymph node, where *S*. Typhimurium harbors both replicating and dormant lifestyles that contribute to the dispersal and persistence of *Salmonella* in the host.

Following the formation of the SCV, *S*. Typhimurium shifts gears from the expression of *Salmonella* Pathogenicity Island (SPI)-1 encoded genes to the expression of SPI-2 encoded genes. This transcriptional reprogramming changes the bacterial physiology from the invasion mode into that of intracellular niche formation through the SPI-2 encoded T3SS-2 bacterial effectors (Hautefort et al., 2008). The T3SS-2-mediated SCV maturation is distinguished by the recruitment of early and late endosomal as well as lysosomal markers, RAB5, RAB7 and LAMP1, respectively (reviewed in (LaRock et al., 2015). The maturation of the SCV is coupled with its migration towards the microtubules organizing center (MTOC), where *S*. Typhimurium begins to replicate inside the SCV (**Figure 2**. Top left, maroon). The intravacuolar lifestyle of *S*. Typhimurium is notable for the subversion of the host endocytic network, which has been regarded as the classic paradigm of *Salmonella* infection. The efficient establishment of the SCV as replicative niche requires three key steps, including SCV integrity maintenance, remodeling the SCV composition through endosome recruitment, and translocation of the SCV towards MTOC. During the establishment of the SCV in epithelial cells, a key step is the formation of the *Salmonella* Induced Filaments (SIFs), a highly dynamic membrane network that extends and helps maintaining a replicative niche by allowing vacuolar bacteria to access nutrients from endocytosed material (Garcia-del Portillo et al., 1993; Drecktrah et al., 2006; Rajashekar et al., 2008; Krieger et al., 2014; Liss et al., 2017).

For the maintenance of SCV integrity, Beuzon et al. and Schroeder et al. have reported that two of the injected T3SS-2 effectors SifA and PipB2 antagonistically regulate the SCV integrity (Beuzón et al., 2000; Beuzón et al., 2002; Schroeder et al., 2010). SifA was identified to interact with a set of host proteins on the SCV membrane and the loss of SifA leads to SCV integrity loss, and the release of bacteria to the cytoplasm, first detected at 10h post infection (pi) using electron microscopy (Beuzón et al., 2000; Beuzón et al., 2002; Diacovich et al., 2009; Schroeder et al., 2010). Besides SifA and its associated factors at the later phase of invasion, a study on the SCV proteome during early invasion has established the significance of SCV-ER contact in SCV integrity regulation (Santos et al., 2015). As mentioned before, the dynamic growth and shrinkage of the SCV is an important factor to the initial maturation steps of the SCV. We have shown that perturbating the balance between SVAT formation and fusion with macropinosomes, largely affects the rate at which the SCV ruptures, in a process that is dependent on the effector SopB (Stévenin et al., 2019). The disruption of the SCV and release of *S*. Typhimurium into the host cytosol, activating the inflammatory cascades as well as the autophagy pathway within the infected cell (**Figure 2**. Top left, blue). The damaged SCV membranes can be targeted by host Galectin 8, which is subsequently ubiquitinated and targeted to autophagy via the recruitment of a series of adaptor proteins (Cemma et al., 2011; Wild et al., 2011; Thurston et al., 2012). Besides host proteins, cytosolic *S*. Typhimurium are also ubiquitylated, serving as a trigger for bacterial restriction via autophagy and cellular inflammation (Otten et al., 2021). SopF, a novel T3SS-1 effector (Cheng et al., 2017; van Wijk et al., 2017; Lau et al., 2019; Xu et al., 2019; Xu et al., 2022) was described to take part in the initial steps of SCV stabilization, preventing Galectin 8 recruitment and therefore avoiding autophagy detection (Lau et al., 2019). In addition, SopF blocks LC3 labeling of the SCVs by preventing the interaction between ATG16L1 and the V-ATPase (Lau et al., 2022), together, these activities work simultaneously in maintaining the integrity of nascent SCV membranes. Additional physical parameters of the cytosolic *S*. Typhimurium, including the intracellular localization, dynamics of replication, the onset of division, subcellular motility, and distribution as well as the fates of cytosolic *Salmonella* should be analyzed in detail by dynamic imaging at single-bacterium level in the future.

Despite the detection of host cytosolic access by the *sifA* mutant, a new paradigm highlighting the importance of the *S*. Typhimurium cytosolic lifestyle was only established in 2010 (**Figure 2**. Top left, bacterial cluster). The new paradigm gained extensive recognition onwards as Knodler et al. reported on a subpopulation of wild type *S*. Typhimurium that escapes from the SCV and rapidly replicates with a doubling time of ~20 min in the host cytosol (Knodler et al., 2010). The rapid *S*. Typhimurium growth phenomenon was termed “hyper-replication” and defined as host cell carrying between 50 to 100 bacteria, which is observed in a range of epithelial cells but not in fibroblasts and immune cells (Knodler et al., 2010; Knodler et al., 2014). It is important to note that subtle discrepancies occur among *S*. Typhimurium hyper-replication dependent on the different epithelial cell culture models. Knodler et al. compared four non-polarized epithelial cell lines, HeLa, Caco-2, HCT and HT-29, highlighting that hyper-replication is conserved, while the distribution of bacterial load in individual infected cell appeared to be diverse across cell lines at 7h pi. In HeLa, 20 to 99 bacteria were found in hyper-replicating cells, while such high numbers were not seen in the other three cell lines (Knodler et al., 2014). Detailed quantitative analysis of cytosolic hyper-replication performed by Fredlund et al. indicates that the rapid growth started instantly upon cytosolic access in about 50% of the cytosolic population, while the other half was targeted for autophagy (Fredlund et al., 2018). Together, these studies suggest that the cytoplasmatic environment of the infected cell is potentially an essential cue for the intracellular behavior of *S*. Typhimurium.

Recent works have enriched our understanding of the characteristics and the regulators on those *S*. Typhimurium that take the host cytosol as their replication niche. They are invasion-primed as determined by the reactivation of SPI-1 encoded genes in the host cytosol, and it was proposed to facilitate the spread to neighboring cells (Knodler et al., 2010; Finn et al., 2017). Both, host factors and bacterial effectors were found to promote hyper-replication. The host autophagy machinery is essential in surveilling the host cytosol and restricting intracellular *S*. Typhimurium growth, such that loss of autophagy components leads to rapid *S*. Typhimurium intracellular growth (Steele-Mortimer et al., 2002; Birmingham et al., 2006). In contrast, another report proposed a positive function of autophagy on *Salmonella* growth, considering the difference between damaged SCVs and cytosolic *S*. Typhimurium (Yu et al., 2014). To handle the contrasting findings, we must note the difference in the experimental approaches used by the two teams, where Steele-Mortimer et al. interrupted autophagy by Wortmannin treatment and Yu et al. knocked down several autophagy genes. Other than the autophagy machinery, Santos et al. reported the facilitating role of the COPII complex for SCV destabilization through mediating the SCV-Golgi interactions, yet the underlying mechanism remains to be revealed (Santos et al., 2015). Aside of the host machinery, SopB, a T3SS-1 effector is injected by cytosolic *S*. Typhimurium in a translocon-independent manner, subverting the host survival regulator Akt and in turn provides a longer window for hyper-replication (Finn et al., 2017). Moreover, SopF acts antagonistically to SopB in modulating membrane dynamics highlighting the need for a precise balance between effectors to maintain the SCV (Lau et al., 2022). Beside these bacterial effectors, a number of *Salmonella* genes were reported to shift the proportion between vacuolar and cytosolic *S*. Typhimurium (Wrande et al., 2016). Recently a transcriptomic analysis revealed a group of genes upregulated in the cytosolic subpopulation indicating that *S*. Typhimurium is exposed to oxidative stress and iron and manganese deprivation in the cytosol (Powers et al., 2021). Even though some hyper-replication regulators have been identified, the precise characteristics and regulatory mechanisms remain largely unknown. Moreover, most of these studies used end-point bulk approaches, not providing insights on the dynamics of the process.

The diverged intracellular niches of *Salmonella* observed in epithelial cells serve as different signals to the host that lead to different consequences. The maturation and remodeling of SCV scavenges a large variety of materials and nutrients within the host, which leads to stress development and the activation of the apoptosis pathways. Apoptotic death of epithelial cells is delayed by the bacterial effector SopB that sustains the activation of the pro-survival Akt pathway (Knodler et al., 2005). A recent report has also suggested the linear ubiquitination on the surface of cytosolic *S*. Typhimurium as a platform for activating the nuclear factor κB (NF-κB) pathway that subsequently restricts the *S*. Typhimurium growth (van Wijk et al., 2017). More recently, ubiquitination of a non-proteinaceous structure, the lipopolysaccharides (LPS), was described. In this work, Otten et al. elegantly demonstrated that the lipid A is tagged by the E3 ubiquitin ligase ring finger protein 213 (RNF213) to restrict bacterial cytosolic proliferation (Otten et al., 2021). Cytosolic *Salmonella* exposes the bacterial LPS, activating the host inflammatory signaling cascades, subsequent cytokine processing, ultimately causing the extrusion and necrotic cell death of the host cell carrying cytosolic *S*. Typhimurium (**Figure 2**. Bottom left) (Knodler et al., 2010; Shi et al., 2014; Yu et al., 2014). The epithelial extrusion and necrotic cell death were proposed to be beneficial for the subsequent bacterial spreading at the tissue level (Knodler et al., 2010). In addition, cytosolic *S*. Typhimurium induces the activation of non-canonical inflammasomes and pyroptosis in response to LPS, in a process relying on interferon-induced guanylate-binding proteins (GBPs) that recruits caspase-4 to the bacterial surface (Santos et al., 2020).

More recently, our group identified for the first time another lifestyle of *S.* Typhimurium in human epithelial cells, a non-growing or dormant subpopulation (Luk et al., 2021). This population was detected in HeLa and polarized Caco-2 cells and it is localized in a vesicular host compartment that lacks most of the known SCV markers and it is not targeted by the host autophagy machinery. In contrast with the majorly viable but not cultivable nature of dormant *S*. Typhimurium described in murine macrophages (Helaine et al., 2010; Helaine et al., 2014), this subpopulation in epithelial cells is viable, less susceptible to antibiotics and can exhibit a delayed expression of SPI-2 when reactivated. Furthermore, the regulation of the formation of this dormant subpopulation depends on the (p)ppGpp stringent response (Luk et al., 2021). It remains to be investigated what are the characteristics of this dormant compartment and what is the role of other bacterial and host factors for the generation of this specific subpopulation.

## Infection of fibroblasts by *S*. Typhimurium

Fibroblasts are the second cell type susceptible to *S*. Typhimurium infection, they are encountered within the lamina propria lying beneath the intestinal epithelial monolayer (**Figure 2**. Bottom middle). Similar to epithelial cell, fibroblasts are non-phagocytic cells that are incapable of active uptake of large particles, such as bacterial pathogens. To understand the invasion process of fibroblasts by *S*. Typhimurium, a couple of cell lines have been used, including mouse embryonic fibroblasts (MEF), mouse 3T3-Swiss albino fibroblasts (3T3 fibroblast), normal rat kidney fibroblasts (NRK-49F) and immortalized human foreskin fibroblasts (BJ-5ta) (**Table 1**). Following the same principle for epithelial cell models, these cell lines have been selected for their suitability for the biological question and compatibility to the experimental setup. However, unlike the catalogue of epithelial cells being used, most of the fibroblast cell lines used for *S*. Typhimurium infection are not derived from human. The MEF is a well-characterized and widely applied fibroblast model with low stringency on genetic manipulation. The 3T3 fibroblast is derived from Swiss albino mouse embryo, which differs in its genetic background and immortalization protocol when compared to MEF. Such differences could potentially account for the variations between the cell lines, for example a weaker intracellular immunity in MEFs. The NRK-49F provides a lower cytotoxicity upon bacterial infection, which enables the prolonged monitoring of invading *S*. Typhimurium, allowing the investigation of intracellular persisting bacteria. Yet, the genetic background of NRK-49F is more distant from that of MEFs or 3T3 fibroblasts. The BJ-5ta is a human-derived immortalized fibroblast cell line that is used in protein expression for *in vitro* experiments, and it provides potentially a better representation of human systems comparing to murine-derived cell lines. Two concerns need to be taken into account with regards to the usage of the fibroblast models. First, the fibroblasts are grown to form a monolayer under *in vitro* cultivation, which does not represent the endogenous anatomic arrangement and composition of cells in the gut lamina propria. Second, the widely used fibroblast cell lines have different origins, which makes the comparison of results across the different model systems difficult, in particular, salmonellosis manifests distinctly in murine and human hosts.

Despite sharing the non-phagocytic feature between epithelial cells and fibroblasts, the behavior of *S*. Typhimurium within the two cell types are remarkably distinct with regards to their subcellular localization and replication rate. *S*. Typhimurium enters both epithelial cells and fibroblasts in a T3SS-1-dependent manner. However, a *S*. Typhimurium SPI-1 mutant maintains up to 30% of its ability to invade fibroblasts, indicating that in this cell type the pathogen is able to induce its internalization in a T3SS-independent manner (Aiastui et al., 2010). Upon internalization, *S*. Typhimurium is contained inside the fibroblasts within an SCV and simultaneously switches from T3SS-1 to the T3SS-2 to drive intra-vacuolar lifestyle progression. It would be interesting to isolate the fibroblast derived SCVs similarly to the protocols established in epithelial cells (Santos et al., 2015).

Despite the similarities of being localized within a vacuolar compartment for bacterial replication in both, epithelial and fibroblast cells, the fibroblast SCV was found to be a different cocoon for *S*. Typhimurium survival based on its distinct morphology. In epithelial cells, extended SIF networks were observed around the SCV after 6h pi, however, such extensive tubular networks were not observed in the vicinity of the fibroblast SCV (Martínez-Moya et al., 1998). Instead, *S*. Typhimurium replicates within or close to a LAMP1-positive aggregate-like structure, termed “aggresome”. The formation of the aggresome is dependent on the T3SS-2 apparatus to maintain SCV integrity and to promote SCV maturation. The aggresome is targeted and degraded by the host aggrephagy together with *S*. Typhimurium from 12 to 24h pi, whereas *S*. Typhimurium dissociated from the aggresome can become persisters (**Figure 2**. Top middle) (López-Montero et al., 2016). The formation of non-replicating *S*. Typhimurium persisters in fibroblasts requires the regulation of the SPI-2 encoded genes differentially regulated by both, the PhoP-PhoQ and the EnvZ-OmpR two-component systems (Núñez-Hernández et al., 2014). PhoP also has been found to control the expression of peptidoglycan hydrolase EcgA in epithelial cells and fibroblasts (Rico-Pérez et al., 2016). Moreover, the peptidoglycan of these non-proliferating bacteria seems to be edited inside the host cell to contain atypical crosslinked muropeptides that contribute to avoiding NF-κB activation and attenuate the pro-inflammatory signaling (Castanheira et al., 2017; Hernández et al., 2022). Very recently, the role of iron acquisition systems was found to be relevant for *S*. Typhimurium survival during persistent infections in fibroblasts. Iron deprivation limits bacterial replication in fibroblasts but not epithelial cells, and when both the Feo and Sit systems are absent, there is a strong decrease in viability of persisting *S*. Typhimurium (Domínguez-Acuña & García-del Portillo, 2022). The non-replicative Salmonellae are also linked to the differential secretion of a set of expressed T3SS-2 effectors that potentially modulates the bacterial dissociation from the aggresome (Núñez-Hernández et al., 2014). It would be important to characterize the persistent *S*. Typhimurium subpopulation within fibroblasts, the molecular regulators and pathophysiological implications.

In spite of the similarities in disease initiation of *Salmonella* invasion, the fibroblast cytosol does not appear to be a permissive replicative niche for *S*. Typhimurium compared to epithelial cells. It is of note that *sifA* mutant within infected fibroblasts do not start to grow rapidly even though they show loss of SCV integrity (Beuzón et al., 2002). When fibroblasts are challenged by wild type *S*. Typhimurium, it was found that 10-20% of the SCVs are ruptured in a T3SS-1-dependent manner, yet no significant cytosolic growth could be detected. Moreover, by carefully tracing the non-hyper-replicating cytosolic *S*. Typhimurium, it was observed that they could slowly replicate in MEFs but not in 3T3 fibroblasts, establishing a link with the low expression of the inflammasome gene Caspase-11 (Thurston et al., 2016b). The inflammatory Caspase cascade was also found to inhibit *S*. Typhimurium cytosolic growth through an uncharacterized mechanism independent of the SCV integrity and caspase-mediated host cell death (Thurston et al., 2016a). To further understand the decision on the subcellular localization of the *S*. Typhimurium replicative niches, a genetic screen was performed to identify the host factors that control the SCV maturation upon internalization. The hits from the screen indicated constituents of the autophagy machinery as key players for successful SCV maturation, and that a loss of autophagy leads to extensive *Salmonella* replication in the MEF cytosol. Kreibich et al. further demonstrated the role of autophagy in repairing the damaged SCV that fostered SCV maturation instead of cytosolic replication (Kreibich et al., 2015). This finding also suggests the dual role of autophagy in SCV integrity maintenance and growth restriction of cytosolic *S*. Typhimurium.

## Infection of immune cells by *S*. Typhimurium

The third invasion-prone cell type towards *S*. Typhimurium infection are the varying immune cells encountered during the infection process that include macrophages, dendritic cells, and neutrophils. The different immune cell types hold distinct and essential defensive roles against pathogen invasion, yet at the same time they can serve as the vector for the systemic spread of *S*. Typhimurium throughout the host (**Figure 2**. Bottom right). To understand the infection process of *S*. Typhimurium in immune cells, some mouse- and human-derived macrophage cell culture models have been employed, they include mouse RAW 264.7 and J774A.1 macrophages, bone marrow-derived macrophages (BMDM), immortalized BMDMs, THP-1 and human monocyte-derived macrophages (hMDMs) (**Table 1**). These cell types have been selected because of their biological features and their amenability to be genetically modified. RAW 264.7 cells are SV40 transformed mouse peritoneal macrophages derived from a single clone, and they have unlimited passage ability. Even though RAW264.7 can be easily cultured, they display a lack of function in certain inflammatory cascades (Pelegrin et al., 2008). RAW264.7 cells are used to study the function of SPI-2 genes as they still allow minor replication of SPI-2 mutants. J774A.1 cells are derived from a tumor in a female BALB/c mouse. The release of inflammatory cytokines is differentially regulated in the two murine macrophage cell lines, therefore offering complementary information to the scientific findings (Hirano et al., 2017). The BMDMs are derived from the mouse bone marrow under suitable *in vitro* culture conditions; consequently, BMDMs provide a closer representation of the endogenous macrophage. It is also important to consider their polyclonal nature, mouse strain specificity and their passage limit. Unlike RAW264.7 cells, BMDMs display a strict restriction for SPI-2 mutant replication. iBMDMs are generated by immortalizing BMDMs through retrovirus infection to bypass the passage limit but introducing functional heterogeneity at the same time. BMDMs and iBMDMs are very versatile since they can be easily derived from genetically modified laboratory mice. THP-1 cells are obtained from a human monocyte cell line originated from an acute monocytic leukemia patient that could be differentiated into macrophage-like cells. hMDMs are derived from monocytes in human blood samples that harbor a great diversity in the genetic background of the donors. Lathrop and colleagues characterized the effect of different hMDMs polarization in *S*. Typhimurium replication, showing that bacteria could only replicate in M0 (non-activated) and M2-activated macrophages, while M1-activated cells killed intracellular *S*. Typhimurium (Lathrop et al., 2015). A more recent study in BMDMs highlighted the metabolic reprograming of these immune cells by *S*. Typhimurium, leading to succinate accumulation which in turn induced the expression of SPI-2 genes. Moreover, it induced antimicrobial peptide resistance in a PmrAB-dependent but PhoPQ-independent manner (Rosenberg et al., 2021). THP-1 is selected as a model for human macrophages for its tractable genetic background and lower restriction on the ethical concerns. However, it should be considered that THP-1 cells are only macrophage-like cells that partially mimic the macrophage phenotypes and their behavior varies depending on the differentiation protocol used. As macrophages have been adopted as a major *in vitro* immune cell culture model to decipher features of *Salmonella* invasion, our discussion below focuses on the macrophage models, unless specified differently.

*Salmonella*-susceptible professional immune cells are phagocytic, which enables dual entry routes of the pathogen through active or passive machineries. This contrasts with the interaction of *S*. Typhimurium with the epithelial cells or fibroblasts. First, *S*. Typhimurium can passively enter the macrophage simply by being phagocytosed within a phagosome. Second, *S*. Typhimurium can also actively enter macrophages by injecting the T3SS-1 effectors, which induces membrane ruffling and bacterial uptake that morphologically resembles the uptake within epithelial cells and fibroblasts. *S*. Typhimurium entering through the action of the T3SS-1 machinery is localized in a host endocytic compartment expected to have a distinct identity and composition from the phagosome containing passively engulfed bacteria. Drecktrah et al. have described the distinct features of the two routes of entry, including the modes of intracellular survival, SCV maturation, as well as the virulence gene expression from SPI-1 and SPI-2 (Drecktrah et al., 2006). In this study, the two uptake routes could be separated by altering the growth of *S*. Typhimurium, where T3SS-1-mediated and phagocytic uptake are achieved by growing *S*. Typhimurium to late-log phase and stationary phase respectively. This experimental setup is not very robust as the expression profile of *S*. Typhimurium changes with the bacterial growth phase, not easily allowing the determination of the two distinct phenotypes are contributed specifically by the different growth stages or the routes of uptake. Moreover, RAW264.7 and J774A.1 macrophages infected with *S*. Typhimurium are rapidly killed by pyroptosis, an inflammatory cell death that depends on caspase-1 (Brennan and Cookson, 2000; Fink and Cookson, 2006; Fink and Cookson, 2007). On the other hand, in THP-1 macrophages, *S*. Typhimurium shows very little intracellular growth, especially when compared to the human adapted serovar *S*. Typhi (Sly et al., 2002).

Upon the *S*. Typhimurium entry through the different routes, the bacterium is enclosed within the SCV that shares some similarities in its composition with the entry compartments in epithelial cells and fibroblasts. Nevertheless, the intracellular lifestyles of the enclosed *S*. Typhimurium differ in these SCVs. A unique feature of *S*. Typhimurium within macrophage SCVs is their replication rate. In 2010, Helaine et al. used a fluorescent dilution reporter to identify the non-replicating population of *S*. Typhimurium within macrophage SCVs (**Figure 2**. Top right). This population was termed as “persisters” that could acquire dormancy and persist in macrophages for a long period while remaining metabolically active and responsive to extracellular stimuli (Helaine et al., 2010). This dormant *S*. Typhimurium subpopulation is clinically significant as the majority of antibiotics target the central cellular machinery in dividing bacteria, which is less available in non-dividing bacteria. Furthermore, the same team revealed that the decision between dormancy and replication is made within the first 30 minutes of infection as well as the diverse metabolic activities among persister bacteria (Helaine et al., 2014). Concerning this, the toxin-antitoxin (TA) system in *S*. Typhimurium was identified as the key player in the decision process, as well as promoting tolerance against stresses including pH and antibiotics that are critical for bacterial persistence (Helaine et al., 2014; Silva-Herzog et al., 2015). The molecular mechanism for one of the key TA systems for dormancy has recently been solved, showing a halt in *S*. Typhimurium protein translation that is linked to the beginning of bacterial dormancy (Cheverton et al., 2016; Rycroft et al., 2018). More recently, it was determined that this nongrowing subpopulation is able to translocate T3SS-2 effectors into the host cell by suppressing the M1 bactericidal response and reprogramming macrophages towards a M2 polarization therefore promoting long term bacterial survival (Stapels et al., 2018).

Besides the dual lifestyles inside the SCV that was described above, *S*. Typhimurium can also gain access to the macrophage cytosol; however, the macrophage cytosol appears to be a non-permissive niche. In line with the epithelial cell and fibroblast models, the *S*. Typhimurium *sifA* mutant escapes into the macrophage cytosol and displays a defected growth phenotype (Beuzón et al., 2000). Once the integrity of the SCV is lost, pathogen associated molecular patterns (PAMPs), such as the LPS of the *S*. Typhimurium surface are exposed to the PAMP receptors in the macrophage cytosol, eliciting a series of local immune responses. Extensive work has been done to understand intracellular immune responses against cytosolic LPS, including the activation of the involved inflammatory pathways and the respective physiological outcomes (Hagar et al., 2013; Shi et al., 2014). Drawing a link between *S*. Typhimurium infection and the pathophysiological outcomes has recently been done by analyzing the major inflammatory cascades: Taking advantage of the cytosolic access property of the *sifA* mutant in iBMDMs, it was found that the key component of the non-canonical inflammatory cascade, Caspase-1 and 11, act to restrict the cytosolic growth of *S*. Typhimurium through a pyroptosis-independent pathway (Thurston et al., 2016b). At the same time, cell death-dependent pathways responsible for the restriction of *S*. Typhimurium cytosolic growth were described, however the molecular mediators and mechanisms of these pathways have not been completely resolved (Thurston et al., 2016b).

Lytic cell death of the *Salmonella*-infected host cell is an effective way to limit the systemic bacterial spread by releasing inflammatory cytokines and recruiting professional immune cells for clearance of cellular remnants. *S*. Typhimurium infection activates the NF-κB pathway that induces necroptotic cell death through the mixed lineage kinase domain-like protein (MLKL) cascade. It was found that the SPI-2 bacterial effectors SseK1 and SseK3 inhibit the MLKL-mediated cell death through GlcNAcylation of different host proteins to counteract the host defense (Günster et al., 2017). Lytic cell death of the infected host cells should be seen as a double edge sword for both, the host as well as *S*. Typhimurium, as lytic cell death releases a myriad of inflammatory cytokines and danger signals that boost the humoral immunity, but also simultaneously releases the intracellular bacteria. A recent report has described a novel mechanism that retained the bacteria but allowed the cytokine release after pyroptotic cell death, termed as pore-induced intracellular traps (PITs). It was found that the pyroptotic corpses trap the *S*. Typhimurium within the cell remnants without killing and recruit the neutrophils for cell corpse clearance (Jorgensen et al., 2016; Hiyoshi et al., 2022).

## Conclusion and outlook

Over the last twenty years, we have obtained an incredible amount of insights and understanding of the host-*Salmonella* interaction during infection through different cell culture models and gene perturbation experiments. This has identified the key molecular cascades involved in driving the intracellular niche formation of this pathogen. Nevertheless, as reviewed here it has also become clear that *Salmonella* dwells within distinct intracellular niches. These niches are different depending on the infected host cell type, but also depending on the chosen model cells and the experimental conditions. Sometimes, the same cell contains bacteria within different niches or within the same niche, but with different physiological behaviors.

Therefore, the described cell culture models have only a limited representation of the endogenous events occurring during the infection process, as endogenous tissues are organized in three dimensions with a broad and complex composition of cells, which are further interconnected with other systems and organs; for example, the gut has resident immune cells constantly surveying the tissue as well as recruited immune cells influx during infection.

The main challenge of the next years will be filling in the gaps in terms of the dynamics of the infection process and going from end-point analysis to single-cell and single-bacterium studies (reviewed in (López-Jiménez and Mostowy, 2021)). Even more, it will become crucial to generate more robust cellular and multicellular models that are physiologically relevant and compatible with live imaging and omics approaches. Finally, to bridge the understanding granted from tissue culture models, *in vitro* models such as organoids and organ-on-a-chip (see **Text Box 1**) and *in vivo* models are valuable assets for discerning our current knowledge and at the same time they open the opportunity to probe biological questions in a more natural and complex model system. This requires a streamlined integration of technologies that provide information on multiple scales, from the subcellular organelle level to the complex organization of an infected organ. *In situ* and *in vivo* imaging allows the tracing of these events in combination with functional markers for specific pathogen localizations and intracellular bacterial growth. The combination of these approaches will decipher major questions of intracellular niche formation of *S*. Typhimurium, for example how the pathogen reaches the host cytosol through SCV membrane damage, which bacterial genes allow the rapid growth within the host cytosol, or which factors drive the “awakening” of the persisting intracellular *Salmonella*.

Text Box 1 **Towards the next generation of biological models.**

The presented conventional cellular models as well as animal models have been powerhouses for generating our current knowledge on *S*. Typhimurium interactions with the host. However, both of them cannot be linked easily to provide a satisfactory and comprehensive physiological representation, while remaining accessible for experimental interventions. Desirable intermediate models bridging the cellular scale with tissue and full body scale are not only demanded by academic scientists to close our knowledge gap, but also by the pharmaceutical industry to obtain more reliable drug screening data prior to later phases of animal and clinical trials.Recently, biologists and bioengineers have harnessed the knowledge in multicellular tissue culture and advanced micro-fabrication techniques to establish *in vitro* models that better recapitulate the *in vivo* environment, including the cellular micro-environment, biomechanical forces and multicellular interactions. Dwelling in endogenous tissues, cells are subjected to different degrees of cell constraints as compared to unconstrained cells grown on plates, which alters cellular physiology, including interactions between the host cell and *S*. Typhimurium (Vonaesch et al., 2013). Endogenous cells also experience various rhythmic compression, stretching and shearing forces from internal cyclical motions (breathing, peristalsis, muscle contraction, blood circulation) or external forces (movements, impacts, weight bearing). Such forces could partially be recapitulated by specifically designed organ-on-chip systems (review by Thompson et al. 2020). Another very important feature of a given endogenous tissue is its 3D multicellular organization, with organoid systems and organ-on-chip models enabling the recapitulation of the endogenous cellular organization and polarity-driven functions via self-organization of cells or compartmentalization. The bioengineering community is also envisioning human-on-chip systems that simultaneously controls multiple organ-on-chip to recapitulate organ-organ interactions in disease modelling and drug discovery (Ingber, 2022). The in-depth discussion on the development and application of these novel multicellular systems has been recently discussed (Yin and Zhou, 2018; Puschhof et al., 2021; Ingber, 2022; Leung et al., 2022).

## Author contributions

CHL wrote the first draft of the manuscript. CV and JE wrote sections of the manuscript. All authors contributed to manuscript edition, read, and approved the submitted version.

## Funding

CHL is part of the Pasteur - Paris University (PPU) International PhD Program. This project has received funding from the European Union's Horizon 2020 research and innovation programme under the Marie Sklodowska-Curie grant agreement No 665807. CHL is a recipient of the Doctoral Fellowship from the Croucher Foundation and 4th year Doctoral Award from Fondation pour la Recherche Médicale. JE acknowledges the support from the European Union (ERC-CG-grant: EndoSubvert), and the ANR (program: HBPSensing and PureMagRupture). JE is part of the IBEID and the Milieu Interieur LabExes.

## Conflict of interest

The authors declare that the research was conducted in the absence of any commercial or financial relationships that could be construed as a potential conflict of interest.

## Publisher’s note

All claims expressed in this article are solely those of the authors and do not necessarily represent those of their affiliated organizations, or those of the publisher, the editors and the reviewers. Any product that may be evaluated in this article, or claim that may be made by its manufacturer, is not guaranteed or endorsed by the publisher.
